# HIV-associated neurological infections in a Brazilian tertiary care center: clinical-epidemiological features and predictors of in-hospital mortality

**DOI:** 10.1590/S1678-9946202668008

**Published:** 2026-01-30

**Authors:** Laísa Rivas Dapousa Ramos, Daniel Ayabe Ninomiya, Murilo Freua Sequeira, Olavo Henrique Munhoz Leite, Marcello Mihailenko Chaves Magri

**Affiliations:** 1 Centro Universitário FMABC, Disciplina de Infectologia, Santo André, São Paulo, Brazil; 2 Universidade de São Paulo, Faculdade de Medicina, Hospital das Clínicas, Departamento de Moléstias Infecciosas e Parasitárias, São Paulo, São Paulo, Brazil

**Keywords:** HIV, Opportunistic infections, Antiretroviral therapy, In-hospital mortality, CNS infections

## Abstract

Neurological manifestations remain a significant cause of hospitalization and in-hospital mortality among people living with HIV (PLWH), even in the era of antiretroviral therapy (ART). This study aims to describe the clinical and epidemiological profile of PLWH with neurological opportunistic infections (nOIs) and to identify factors associated with in-hospital mortality. We conducted a retrospective cohort study with PLWH aged >18 years hospitalized due to nOIs between November 2017 and December 2021 at a tertiary hospital in Brazil. Demographic, clinical, and laboratory data were extracted from electronic medical records. Logistic regression was used to evaluate associations between patient characteristics and in-hospital mortality. Among 237 hospitalized PLWH, 89 (37.6%) had nOIs. The median CD4 count at admission was 55 cells/mm³ (IQR 22.5–149), and 91.7% had previously used ART (only 22.7% used it regularly). The most frequent infections were cerebral toxoplasmosis (50.6%), cryptococcal meningitis (10.1%), and progressive multifocal leukoencephalopathy (9%). A total of 19 in-hospital deaths occurred. In the multivariate analysis, undefined neurological infections (aOR: 8.67; 95%CI: 1.23–61.17) and ICU admission (aOR: 58.61; 95% CI: 10.24–335.49) were independently associated with mortality. In conclusion, severe immunosuppression and low ART adherence were common in this cohort. Cerebral toxoplasmosis was the most prevalent neurological infection. ICU admission and undefined neurological syndromes were strong predictors of in-hospital mortality. Early diagnosis, prompt treatment, and strategies to improve ART adherence are essential to reduce fatal outcomes in this population.

## INTRODUCTION

Neurological complications are a well-recognized and frequent cause of morbidity and mortality among people living with HIV (PLWH) since the onset of the AIDS epidemic. These manifestations may arise from direct viral effects on the central nervous system (CNS), immune reconstitution inflammatory syndrome (IRIS), or opportunistic infections (OIs) that occur in the setting of advanced immunosuppression^1-7^.

Although the introduction of combination antiretroviral therapy (ART) has significantly reduced the incidence of many OIs, CNS involvement remains a major clinical concern in low- and middle-income countries (LMICs), especially where delayed diagnosis and poor ART adherence persist^1,5,6,8,9^. The most common HIV-associated neurological OIs include cerebral toxoplasmosis, cryptococcal meningitis, progressive multifocal leukoencephalopathy (PML), tuberculous meningitis, cytomegalovirus (CMV) encephalitis, and neurosyphilis^2-9^.

In Brazil, the universal availability of ART since the mid-1990s has led to significant public health achievements^10,11^. However, late presentation, irregular follow-up, and ART discontinuation still contribute to high rates of AIDS-related complications, including CNS infections^12,13^. A better understanding of clinical and epidemiological profiles of PLWH with neurological OIs, as well as the identification of factors associated with in-hospital mortality, is crucial to guide effective interventions in this population. This study aims to describe clinical and epidemiological features of PLWH hospitalized with CNS opportunistic infections in a Brazilian tertiary hospital and to identify predictors of in-hospital mortality.

### Ethics

The study was approved by the local ethics committee (CAAE Nº 59481822.0.0000.0082), and informed consent was waived due to the retrospective design and use of anonymized data.

## MATERIALS AND METHODS

We conducted a retrospective cohort study at Hospital Estadual Mario Covas in Santo Andre, Sao Paulo State, Brazil. This tertiary referral university hospital serves a catchment population of approximately 2.8 million people across seven municipalities in the Greater ABC region, providing specialized care in high-complexity areas. Medical records of HIV-positive adults (≥18 years) hospitalized with neurological manifestations between November 2017 and December 2021 were reviewed.

Patients were identified using International Classification of Diseases (ICD-10) codes associated with CNS opportunistic infections (OIs): B24, B58.9, B58.2, B45.7, B45.1, B25.9, B25, A52.3, A17, A81.2, A81.9, G03.9, and G04.9. Inclusion criteria were a confirmed HIV diagnosis, hospitalization during the study period, and clinical and/or laboratory evidence of CNS OI. Exclusion criteria included neoplastic CNS involvement (e.g., primary CNS lymphoma), immune reconstitution inflammatory syndromes, or insufficient clinical data.

Data were collected using REDCap (Research Electronic Data Capture; Vanderbilt University, Nashville, TN, USA). According to standard criteria^2,3-6,8,14^, OIs were defined as either presumptive or confirmed diagnoses for the following: Cerebral toxoplasmosis – compatible clinical and radiological findings, clinical and radiological improvement after 14 days of anti-*toxoplasma* therapy, and absence of other causes of expansive brain lesions. Cryptococcal meningitis – compatible clinical findings and positive cerebrospinal fluid (CSF) tests for *C. neoformans* (India ink, antigen latex test, or culture). Tuberculous meningitis – compatible clinical manifestations, CSF cytological changes, positive CSF tests for *M. tuberculosis* (PCR, TB-RMT, culture, pBAAR), or compatible ADA values, with clinical improvement after antituberculosis therapy. PML – compatible clinical and radiological findings, with no evidence of other causes of non-expansive brain lesions. Cytomegalic disease categorized as encephalitis, retinitis, or polyradiculopathy. Encephalitis – compatible clinical and radiological manifestations, CSF cytological changes, positive CSF PCR for CMV. Retinitis – compatible clinical manifestations and fundoscopy showing CMV infection, with improvement after ganciclovir. Polyradiculopathy – compatible clinical findings, CSF cytological changes, compatible MRI changes, positive CMV PCR in peripheral blood, stabilization or clinical improvement after ganciclovir and/or foscarnet, absence of other causes of polyradiculopathy. Neurosyphilis – compatible clinical manifestations, reactive treponemal and non-treponemal serum tests, clinical improvement after treatment, and absence of other causes of meningitis. Neurological infection without defined etiology – PLWH presenting meningitic or encephalitic syndrome at admission, after exclusion of non-infectious neurological differential diagnoses. Other opportunistic infections included oral, esophageal, tracheal, bronchial, or lung candidiasis; herpes zoster (≥2 episodes or ≥2 dermatomes); listeriosis; *Pneumocystis jiroveci* pneumonia; extrapulmonary tuberculosis; Kaposi's sarcoma; cytomegalovirus disease (excluding liver, spleen, lymph nodes, or nervous system involvement); intestinal cryptosporidiosis; intestinal isosporiasis; disseminated mycoses; reactivation of Chagas disease (meningoencephalitis and/or myocarditis); disseminated atypical leishmaniasis; and bacillary angiomatosis.

Baseline characteristics included demographic, epidemiological, and clinical variables. Demographic data comprised age, sex, self-reported race/skin color, sexual orientation, geographic origin, educational attainment, and history of substance use, including tobacco, alcohol, and illicit drugs. HIV-related epidemiological variables included prior opportunistic infections, CD4+ T-cell count and plasma HIV RNA levels at admission, previous awareness of HIV status, and history of ART therapy, categorized as regular, irregular, or discontinued use. The categorization of ART adherence was based on medical records, patient and family reports, and, mainly, data from the Laboratory Test Control System of the National Network for CD4/CD8 Lymphocyte Count and HIV Viral Load (SISCEL), a digital database implemented in Brazil in 2012. Because exact dates of the last ART dose/dispensing were inconsistently documented across self-reports, clinician notes, and pharmacy records, we could not derive a reliable ‘time since ART interruption’ variable. In addition to recording lymphocyte counts and viral loads, SISCEL provides historical data on antiretroviral regimens, prescription validity, prescribing physician, medication dispensing frequency, and responsible pharmacy. The type and number of central nervous system (CNS) opportunistic infections were also recorded. Additional epidemiological data included the presence of non-HIV comorbidities. Clinical variables assessed at admission included CD4+ T-cell count, plasma HIV RNA levels, prior ART adherence patterns, classification of CNS infection (with or without defined etiology), number of concurrent CNS infections, presence of non-CNS opportunistic infections during hospitalization, and clinical outcome (discharge or in-hospital death).

The primary outcome was in-hospital mortality. Data were analyzed descriptively using absolute and relative frequencies. Associations between categorical variables were assessed using the Chi-square test or Fisher's exact test, as appropriate. Univariate and multivariate logistic regression analyses were performed to identify factors associated with death. Categorical variables are described as frequencies and percentages. To evaluate the effect of predictor variables (clinical characteristics) on the outcome variable (in-hospital mortality), univariate and multivariate logistic regression models were applied. Variables included in the univariate analysis were age, sex, race, educational level, substance abuse, smoking, alcohol consumption, CD4 T-lymphocyte count (cells/mm^3^), plasma HIV viral load (copies/mL) at admission, prior use of ART, central nervous system infection, opportunistic infection in other organ systems, and ICU admission. For descriptive variables with substantial missing data ("not reported"), percentages for reported categories were calculated using the available-case denominator for that variable, while "not reported" categories were listed separately with the total cohort denominator. In multivariable analyses, we limited covariates to those with minimal missing data and used complete-case analysis; socio-behavioral variables with high levels of missing data were excluded as predictors. Given the relatively small sample size compared to the number of predictors, variables with a significance level of ≤20% in the univariate analysis were selected for inclusion in the initial multivariate model. Variables that did not remain statistically significant at the 5% level were subsequently excluded from the final multivariate model. All analyses were conducted using SPSS statistical software (version 20.0; IBM Corp., Armonk, NY, USA) and Stata statistical software (version 17, StataCorp LLC, College Station, TX, USA), with a two-sided significance threshold of 5%. Given low counts in some exposure strata, we anticipated sparse-data bias and imprecise estimates for selected coefficients. Accordingly, these parameters were interpreted with caution, focusing on the direction and consistency of effects rather than point estimates alone.

## RESULTS

**Figure 1:**
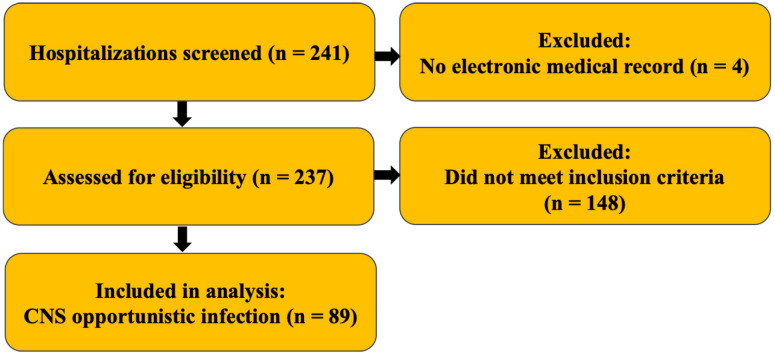
STROBE-style flow diagram of patient selection. Screened (n=241); excluded due to missing electronic medical records (n=4); assessed (n=237); excluded for not meeting inclusion criteria (n=148); included in the analysis with CNS opportunistic infection (n=89). No additional exclusions were made for missing key variables.

A total of 241 PLWHA were hospitalized between November 2017 and December 2021. Four patients were excluded due to lack of electronic medical records, resulting in a final sample of 237 patients. Among these, 40% were diagnosed with gastrointestinal opportunistic infections, 26% with respiratory infections, and 18% with hematological opportunistic infections. Overall, 37.6% (n=89) were diagnosed with at least one opportunistic neurological infection and met the inclusion criteria (Figure 1).

Time since HIV diagnosis to admission varied across etiologies. Median years (range) were: neurotoxoplasmosis, 10 (1–20); neurotuberculosis, 9 (3–15); neurocryptococcosis, 7 (0–24); cytomegalovirus disease, 6.5 (0–22); progressive multifocal leukoencephalopathy (PML), 6 (1–20); and neurosyphilis, 5 (0–16). These distributions indicate that most patients had a long-standing HIV infection, while the lower bound of 0 years in several groups suggests a subset with recent diagnosis or delayed presentation.

Reported substance use was common: 44.3% were current or former illicit drug users, 57.1% were current or former smokers, and 67.6% reported current or prior alcohol consumption (Table 1). Given the small sample size, subgroup analyses—such as distinguishing current from former users or social drinkers from alcohol-dependent individuals—were not performed. Most patients were male (65.2%), self-identified as White (52.9%), and had completed secondary education (41.8%). Regarding gender identity and sexual orientation, 57.3% were cisgender men, and 56.2% identified as heterosexual.

**Table 1:** Sociodemographic, behavioral, and clinical characteristics of all patients admitted from 2017 to 2021

Characteristic		All patients (89) n (%)
**Age (years)**		
	18–20 years	1 (1.1)
	21–30 years	14 (15.7)
	31–40 years	31 (34.8)
	41–50 years	22 (24.7)
	51–60 years	16 (18.0)
	61–70 years	5 (5.6)
**Sex**		
	Male	58 (65.2)
	Female	31 (34.8)
**Race**		
	Non-White (Black or Mixed-race)	41 (47.1)
	White	46 (52.9)
	Not reported	12 (13.5)
**Education**		
	Incomplete primary education	11 (20)
	Complete primary education	19 (34.5)
	Complete secondary education	23 (41.8)
	Complete higher education	2 (3.6)
	Not reported	34 (38.2)
**Region of origin**		
	Sao Paulo	57 (64)
	Other regions of Brazil	30 (33.7)
**Housing status**		
	Stable housing	82 (92.1)
	Institutionalized	0 (0)
	Public space dwelling	3 (3.4)
	Not reported	-
**Gender identity**		
	Cisgender man	51 (57.3)
	Cisgender woman	31 (34.8)
	Transgender man	0 (0)
	Transgender woman	4 (4.5)
	Non-binary	0 (0)
	Not reported	3 (3.4)
**Sexual orientation**		
	Heterosexual	50 (56.2)
	MSM	16 (18)
	Bisexual	0 (0)
	Not reported	23 (25.8)
**Illicit drug use**		
	Current/former user	35 (44.30)
	Does not use	44 (55.7)
	Not reported	10 (11.2)
**Tobacco use**		
	Smoker/former smoker	44 (57.1)
	Does not smoke	33 (42.9)
	Not reported	12 (13.5)
**Alcohol use**		
	Current/former/social drinker	50 (67.6)
	Does not drink	24 (32.4)
	Not reported	15 (16.9)
**Previous comorbidities**		
	Yes	24 (27)
	No	65 (73)
**Previous opportunistic infection**		
	Yes	10 (11.2)
	No	79 (88.8)
**Previous HIV diagnosis**		
	Yes	48 (53.9)
	No	41 (46.1)
**CD4+ T cell count (cels/mm** ^3^)		
	≤50	48 (53.9)
	51–100	8 (9)
	101–200	18 (20.2)
	201–350	4 (4.5)
	351–500	2 (2.2)
	>500	9 (10.1)
**HIV serum viral load** (copies/mL)		
	Undetectable	5 (5.6)
	<50	4 (4.5)
	50–1,000	15 (16.9)
	>1,000	65 (73)
**Previous ART use**		
	Yes	44 (49.4)
	No	45 (50.6)
**ART use**		
	Regular	10 (11.2)
	Irregular	15 (16.9)
	Discontinued	19 (21.3)
	No use	45 (50.6)
**Clinical syndrome on admission**		
	Meningitis	22 (24.7)
	Encephalitis	59 (66.3)
**Diagnostic criteria**		
	Clinical	83 (93.3)
	Radiological	78 (87.6)
	Microbiological	39 (43.8)
**Neurological manifestation**		
	Neurosyphilis	6 (6.7)
	Neurotoxoplasmosis	45 (50.6)
	PML	8 (9.0)
	Neurocryptococcosis	9 (10.1)
	CMV disease	5 (5.6)
	Tuberculosis	4 (4.5)
	Unknown	6 (6.7)
	Mixed infection	6 (6.7)
**Neurological manifestations (n)**		
	Unknown etiology	6 (6.7)
	1	77 (86.5)
	2	3 (3.4)
	3	3 (3.4)
**Other opportunistic infections (non-CNS)**		
	No	43 (48.3)
	Yes	46 (51.7)
**ICU admission**		
	No	67 (75.3)
	Yes	22 (24.7)

PML = Progressive multifocal leukoencephalopathy; ART = Antiretroviral therapy; ICU = Intensive care unit; CMV = Cytomegalovirus.

**Figure 2:**
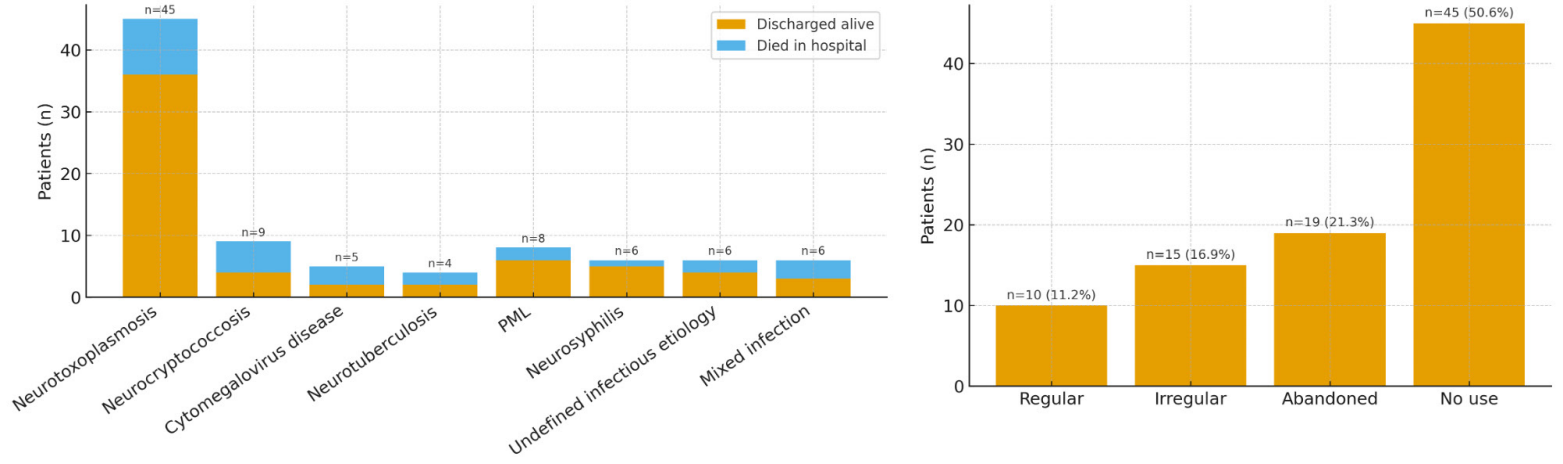
Summary of clinical outcomes and treatment status. Left: clinical outcomes by infection type (discharged alive versus in-hospital death); right: ART use at admission. In-hospital mortality: 21.3% (19/89). Etiology categories are not mutually exclusive; counts reflect total per category.

At admission, 53.9% of patients had a prior HIV diagnosis, and 91.7% had previously initiated ART; however, only 22.7% reported regular use, while 34.1% reported irregular adherence and 43.2% had discontinued therapy (Figure 2). The median CD4+ T-cell count was 55 cells/mm^3^ (IQR: 22.5–149), and 53.9% had <50 cells/mm^3^. HIV RNA was >1,000 copies/mL in 73% of patients, and only 5.6% had undetectable viral load. The most common diagnosis was cerebral toxoplasmosis (45/89; 50.6%), followed by cryptococcal meningitis (9/89; 10.1%), PML (8/89; 9.0%), CMV-related disease (5/89; 5.6%), tuberculous meningitis (4/89; 4.5%), and neurosyphilis (6/89; 6.7%). Six patients (6.7%) presented with undefined CNS syndromes despite complete diagnostic workup. Three patients (3.4%) had two concurrent CNS infections, and another three (3.4%) had three. The majority (86.5%) had a single CNS infection. Most presentations were encephalitic (66.3%), while 24.7% were classified as meningitis. Concurrent non-CNS opportunistic infections were documented in 51.7% of cases, including oral and esophageal candidiasis, pulmonary tuberculosis, *Pneumocystis jirovecii* pneumonia, and disseminated histoplasmosis. ICU admission was required for 24.7% of patients (Table 1).

**Figure 3:**
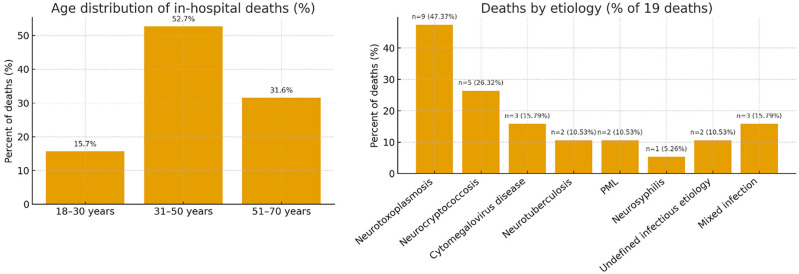
In-hospital deaths (n=19; 21.3%). Left: age distribution of deaths; right: deaths by etiology (% of 19 deaths). Percentages in the etiology panel are calculated as n/19; categories are not mutually exclusive, as patients may have multiple etiologies.

In-hospital death occurred in 19 patients (Figure 3). In the univariate analysis, male sex (p=0.012), number of CNS infections (p=0.035), presence of non-CNS OIs (p=0.030), and ICU admission (p<0.001) were significantly associated with mortality (Table 2). ART discontinuation showed a trend toward significance (p=0.079). Patients with three concurrent CNS infections had the highest mortality rate (66.7%). In the multivariate logistic regression model, only two variables remained independently associated with death: ICU admission (adjusted odds ratio [aOR]: 58.6; 95%CI: 10.2–335.5) and undefined CNS etiology (aOR: 8.7; 95%CI: 1.2–61.2). Parameters with very wide CIs (e.g., undefined etiology: aOR 26.59, 95%CI 1.34–527.25) reflected limited information in specific strata and are consistent with sparse-data bias. The number of concurrent CNS infections did not remain statistically significant after adjustment (Table 3).

**Table 2:** Characteristics and factors associated with in-hospital mortality by univariate analysis at Hospital Estadual Mario Covas (HEMC) from 2017 to 2021.

Characteristic		All patients N=89 (100%)	Survival N=70 (78.7%)	Death N=19 (21.3%)	*p*
Age (median; years)		41,66	43,5	42	0.924*
Male		58 (65)	41 (70.7)	17 (29.3)	**0.012** **
Caucasian		46 (51.7)	38 (82.6)	8 (17.4)	0.421*
**CD4 T-lymphocyte count (cell/mm³)**					0.418*
	≤50	48 (53.9)	34 (70.8)	14 (29.2)	
	51–100	8 (8.9)	8 (100.0)	0 (0.0)	
	100–200	18 (20.22)	14 (77.8)	4 (22.2)	
	200–350	4 (4.5)	4 (100.0)	0 (0.0)	
	350–500	2 (2.2)	2 (100.0)	0 (0.0)	
	>500	9 (10.1)	8 (88.9)	1 (11.1)	
**HIV serum quantitative PCR (copies/ml)**					0.096*
	Undetectable	5 (5.6)	4 (80.0)	1 (20.0)	
	less than 50	4 (4.5)	3 (75.0)	1 (25.0)	
	50–1,000	15 (16.9)	15 (100.0)	0 (0.0)	
	higher than 1,000	65 (73.0)	48 (73.8)	17 (26.2)	
**ART use**					0.309*
	Never used	45 (50.5)	32 (71.1)	13 (28.9)	
	Irregular use	15 (16.9)	14 (93.3)	1 (6.7)	
	Discontinued	19 (21.34)	16 (84.2)	3 (15.8)	
**Neurologic manifestation**					0.057*
	Neurosyphilis	6 (6.7)	6 (100.0)	0 (0.0)	
	Neurotoxoplasmosis	45 (50.6)	38 (84.4)	7 (15.6)	
	Progressive multifocal leukoencephalopathy	8 (9.0)	7 (87.5)	1 (12.5)	
	Neurocryptococcosis	9 (10.1)	6 (66.7)	3 (33.3)	
	Cytomegalic disease	5 (5.6)	3 (60.0)	2 (40.0)	
	Neurotuberculosis	4 (4.5)	4 (100.0)	0 (0.0)	
	Undefined infectious etiology	6 (6.7)	3 (50.0)	3 (50.0)	
**Number of neurological infections**					**0.035** *
	One	77 (86.5)	64 (83.1)	13 (16.9)	
	Two	3 (3.3)	1 (33.3)	2 (66.6)	
	Three	3 (3.3)	1 (33.3)	2 (66.6)	
**Concomitant opportunistic infections (other systems)**		46 (51.7)	32 (69.6)	14 (30.4)	**0.030** **
**ICU admission**		22 (24.7)	5 (22.7)	17 (77.3)	**<0.001** *

* Descriptive level of Fisher's exact test;

** descriptive level of the Chi-square test.

**Table 3:** Multivariate logistic regression analysis

Characteristic		Adjusted OR (95%CI)	p-value	Final OR (95%CI)	p-value
**Sex**					
	Male (ref.)	–	–	–	–
	Female	0.11 (0.01–1.31)	–	–	–
**Prior ART use**					
	Yes (ref.)	–	–	–	–
	No	2.07 (0.22–19.07)	0.520	–	–
**Neurological infections**					
	1 (ref.)	–	–	–	–
	>1	3.10 (0.09–103.87)	0.331	–	–
	Undefined infectious etiology	193.16 (3.71–10,047.59)	**0.009**	26.59 (1.34–527.25)	**0.031**
**Concomitant OI (non-CNS)**					
	No (ref.)	–	–	–	–
	Yes	6.01 (0.67–54.03)	0.110	–	–
**ICU admission**					
	No (ref.)	–	–	–	–
	Yes	219.00 (17.74–2,704.35)	**<0.001**	196.77 (20.28–1,909.24)	**<0.001**

OR = odds ratio; CI = confidence interval; ART = antiretroviral therapy; ICU = intensive care unit; OI = opportunistic infection;

* multivariate logistic regression model and Chi-square test. Statistically significant p-values (p<0.05) are shown in bold.

## DISCUSSION

This study highlights the persistent burden of nOIs among hospitalized PLWH in a tertiary care setting. Cerebral toxoplasmosis was the most frequent diagnosis, followed by cryptococcal meningitis, tuberculous meningitis, and CMV-related neurological syndromes. Intensive care unit (ICU) admission and the presence of neurological syndromes of undefined etiology were the strongest predictors of in-hospital mortality. Most patients presented advanced immunosuppression and a history of ART discontinuation or irregular use, despite having prior access to treatment.

The frequent occurrence of co-infections, severe neurological impairment, and diagnostic uncertainty reflects both individual vulnerability and systemic gaps in HIV care. These findings underscore a persistent pattern of advanced disease presentation, profound immunosuppression, and therapeutic disengagement, which is particularly relevant in LMICs. Sustained virologic suppression and immunologic recovery by means of immediate ART initiation, an established paradigm for more than a decade, remain central to the clinical management of PLWH. Nevertheless, ensuring continuity of care and sustained ART adherence continues to be a critical challenge^1-9^. Furthermore, many emergency department clinicians address neurological symptoms without considering nOIs, leading to repeated healthcare visits before an accurate diagnosis and appropriate treatment are established. Nearly half of the cohort were newly diagnosed at admission (41/89; 46.1%), with many presenting with profound immunosuppression (CD4 ≤50: 48/89; 53.9%), indicating late diagnosis as a key contributor of disease severity. Well-structured diagnostic workflows, supported by the training of frontline professionals, are essential to effective management.

Moreover, as observed in our cohort, these diagnoses remain often challenging even for infectious disease specialists, and their management depends on diagnostic tools that are frequently unavailable. The Hospital Estadual Mario Covas, a university hospital within the Brazilian Unified Health System (SUS), operates under structural constraints, including the absence of several point-of-care technologies such as molecular diagnostic panels for meningoencephalitis, lateral flow tests for cryptococcal antigen, and lateral flow assays for lipoarabinomannan. The availability of these resources would render diagnostic workflows not only more efficient but, in many cases, feasible.

In contrast, high-income countries with robust health systems have experienced a significant decline in AIDS-defining OIs. In the NA-ACCORD study^1^, which included over 63,000 PLWH in North America, the incidence of OIs dropped from 3.0 to 1.5 per 100 person-years between 2000 and 2010. This improvement was attributed to early ART initiation, strong linkage to care, and prophylaxis, resources often lacking in LMICs.

Our findings align with global trends. In sub-Saharan Africa, where HIV burden remains high, nOIs contribute substantially to morbidity and mortality among hospitalized PLWH. Howlett (2019) reports that approximately 20% of PLWH present with central nervous system infections, with six- to nine-month mortality rates of 60%–70%, largely due to diagnostic delays arising from limited access to neuroimaging, lumbar puncture, and molecular diagnostics^15^.

Although in-hospital mortality in our Brazilian cohort was lower (21.3%), ICU admission remained the strongest predictor of death (aOR 219.00), echoing findings from African studies in which over 75% of ICU-admitted PLWH with nOIs die. Over half of patients had CD4+ T-cell counts below 50 cells/mm^3^ at admission, and 73% had HIV RNA levels >1,000 copies/mL, reflecting uncontrolled viremia and late-stage HIV. Despite 91.7% having previously initiated ART, only 22.7% reported regular adherence, and 43.2% had discontinued treatment entirely, highlighting systemic barriers to continuity of care and long-term retention, as previously documented in Brazilian cohorts^11-13^.

Late presentation and ART discontinuation are well-established risk factors for AIDS-defining illnesses and poor prognosis^1,16^. Bongomin *et al*.^17^ emphasize these factors as key predictors of AIDS-related morbidity and mortality across African settings. Our data corroborate this pattern, showing that most neurological infections occurred among severely immunosuppressed individuals, many of whom had with a prior HIV diagnosis but were not virologically suppressed.

These findings underscore critical differences in healthcare systems. In sub-Saharan Africa, resource limitations—including restricted access to diagnostic tools, trained personnel, and ICU capacity—amplify mortality risk associated with nOIs. In Brazil, SUS provides broader ART availability and comparatively better-equipped facilities, partially mitigating mortality. However, structural barriers such as insufficient retention strategies and limited point-of-care diagnostics continue to hinder optimal outcomes. Collectively, these observations highlight the need for context-specific interventions that enhance early diagnosis, sustain ART engagement, and provide differentiated care for individuals at high risk of disengagement.

A recent Brazilian study by Telles *et al*.^11^, conducted at another tertiary care center in Sao Paulo, prospectively evaluated 105 hospitalized PLWH with neurological complaints and reported findings strikingly similar to ours. Opportunistic infections remained the leading etiologies, with cerebral toxoplasmosis (36%), cryptococcal meningitis (14%), tuberculous meningitis (8%), and CMV-related CNS disease (12%) as the most frequent diagnoses. Notably, CMV was the third most frequent pathogen, whereas in our cohort it accounted for only 1.1% of cases, possibly reflecting differences in diagnostic access or patient selection.

Telles *et al*.^11^ reported that ICU admission (OR 6.2) and altered level of consciousness (OR 22.7) were independently associated with in-hospital mortality, aligning with our finding that ICU admission was the strongest predictor of death (aOR 219.00). Despite a lower in-hospital mortality rate in their cohort (12% versus 21.3% in ours), one-year all-cause mortality reached 31%, underscoring the sustained risk of poor outcomes even after hospital discharge. This highlights the importance of post-discharge monitoring and continued outpatient engagement, particularly among patients with advanced immunosuppression and complex neurological presentations. While ICU admission emerged as a clear marker of poor prognosis, the specific etiologies underlying CNS disease warrant further consideration, with *Toxoplasma gondii* remaining the predominant cause of neurological morbidity among PLWH in Latin America.

*Toxoplasma gondii* remains a major cause of CNS lesions among AIDS patients, especially in Brazil. In Sao Paulo State, it ranks as the third most common AIDS-defining illness in the HAART era and accounts for the first HIV diagnosis in up to 35% of patients in some cohorts^13,18^. In our study, cerebral toxoplasmosis was the leading diagnosis (50.6%) and, although associated with significant morbidity, it was not an independent predictor of mortality, likely reflecting improved access to empiric treatment protocols and early clinical intervention.

Cryptococcal meningitis remains a global challenge, particularly in LMICs, where it accounts for 10%–20% of AIDS-related deaths. In our cohort, it represented 10.1% of cases, consistent with reports from Brazil and China^11,19^. In African studies, it accounts for up to 62% of adult meningitis cases. The disease was not independently associated with in-hospital mortality in our sample, but its known severity highlights the importance of early CrAg screening in outpatient settings and the need for access to flucytosine and liposomal amphotericin B^16,20^.

Coinfections also contribute to morbidity. Telles and Vidal^18^ reported that 20% of PLWH with cerebral toxoplasmosis had neurological coinfections, most frequently CMV, resulting in prolonged hospitalization (62 versus 30 days), increased ICU admission (44% versus 14%), and a trend toward higher mortality (33% versus 8.6%). In our study, CMV-related syndromes were present in 5.6% of patients, with a high mortality rate (40%). These data underscore the importance of careful monitoring for CNS coinfections in severely immunosuppressed patients.

Tuberculous meningitis (TBM), although less frequent, remains one of the deadliest CNS infections in PLWH. In a Brazilian cohort, Croda *et al*.^16^ reported a nine-month mortality of 41% among TBM patients, with only 15% presenting classical symptoms. In our study, TBM occurred in 6.7% of patients and shared similarly poor outcomes. These results reinforce the need for rapid TB diagnostics and empiric treatment in PLWH presenting meningeal syndromes.

Progressive multifocal leukoencephalopathy (PML) was diagnosed in 9% of patients. Caused by JC virus reactivation, PML is typically associated with profound immunosuppression and has no specific treatment beyond ART-induced immune recovery. Mortality remains high, particularly among patients with delayed diagnosis or irreversible neurological damage. In our cohort, although PML patients had advanced disease, PML was not significantly associated with mortality, likely due to the small sample size. High rates of *Treponema pallidum* co-infection have been reported, particularly among men who have sex with men (MSM). Routine lumbar puncture is essential in patients with neurologic symptoms and positive serology. All neurosyphilis patients in this study survived, likely reflecting the treatable nature of the infection when diagnosed promptly^1-7^.

Six patients (6.7%) had CNS syndromes with no confirmed etiology despite comprehensive diagnostic workup. This subgroup had the highest mortality rate (50%) and was independently associated with death in multivariate analysis (aOR: 193.16). Possible causes include atypical presentations of known pathogens, primary CNS lymphoma, or IRIS. These cases underscore a diagnostic gap in real-world practice, especially in settings with limited access to PCR-based diagnostics, neuroimaging, and biopsy^21,22^. Improved syndromic algorithms and point-of-care diagnostic tools are urgently needed in such contexts.

Diagnostic challenges of CNS infections are further compounded by cases with undetermined etiologies. In our cohort, 6.7% of patients had undefined CNS syndromes despite extensive evaluation, and this group exhibited the highest mortality (50%), with undiagnosed etiologies independently associated with death (aOR: 193.16). Howlett^15^ similarly reported that up to 49% of fatal CNS cases in African cohorts remained undiagnosed at autopsy. These findings emphasize the urgency of expanding access to lumbar puncture, advanced neuroimaging, and molecular diagnostics. Molecular testing has become an essential tool to improve diagnosis in this population. A recent study from Manaus city, Amazonas State, Brazil, demonstrated that 36.7% of cerebrospinal fluid samples from HIV-infected adults with neurological symptoms yielded detectable pathogens by qPCR, with *Toxoplasma gondii, Cryptococcus* spp., and herpesviruses being the most common^22^. Such approaches may reduce the proportion of cases with unknown etiology and guide targeted treatment.

### Limitations

This study presents several limitations. First, its retrospective design is inherently subject to information bias and limits causal inference. Second, the investigation was conducted at a single tertiary referral center, which may hinder the generalization of the findings to other healthcare settings. Third, the relatively small sample size limited the statistical power for detailed subgroup analyses and constrained the evaluation of less frequent etiologies using multivariate models. Some exposure–outcome strata included few observations, leading to wide CIs and potential sparse-data bias. These estimates should, therefore, be interpreted cautiously. Finally, the lack of long-term follow-up precluded assessment of post-discharge mortality and neurological sequelae, which are critical outcomes in this patient population. Although neuroimaging and CSF analyses were routinely performed, the unavailability of brain biopsy or autopsy limited diagnostic clarification in several fatal CNS cases.

## CONCLUSION

This study reinforces the ongoing clinical and public health challenge posed by nOIs in PLWH with advanced disease in LMICs. The predominance of cerebral toxoplasmosis, severity of cryptococcosis, and high proportion of undiagnosed cases highlight the urgent need to strengthen early testing, diagnostic capacity, retention strategies, and access to antifungals and ART. Improving outcomes in this vulnerable population requires the integration of outpatient care, systematic CrAg screening for PLHIV with CD4 counts <100 cells/mm^3^, implementation of protocols for early CNS-OI diagnosis, broader access to molecular diagnostics, and enhanced ART adherence with structured post-discharge follow-up.

## Data Availability

The anonymized dataset generated during this study is available from the corresponding author upon reasonable request.
